# Neural correlates of theory of mind in typically-developing youth: Influence of sex, age and callous-unemotional traits

**DOI:** 10.1038/s41598-019-52261-y

**Published:** 2019-11-07

**Authors:** Yidian Gao, Jack C. Rogers, Ruth Pauli, Roberta Clanton, Rosalind Baker, Philippa Birch, Lisandra Ferreira, Abigail Brown, Christine M. Freitag, Graeme Fairchild, Pia Rotshtein, Stephane A. De Brito

**Affiliations:** 1 Medical Psychological Institute, Second Xiangya Hospital, Central South University, Changsha, Hunan China; 2 Centre for Human Brain Health, School of Psychology, University of Birmingham, Birmingham, UK; 3 Institute for Mental Health, School of Psychology, University of Birmingham, Birmingham, UK; 4 Department of Child and Adolescent Psychiatry, Psychosomatics and Psychotherapy, University Hospital Frankfurt, Goethe University, Frankfurt am Main, Germany; 5 Department of Psychology, University of Bath, Bath, UK

**Keywords:** Cognitive neuroscience, Human behaviour

## Abstract

Theory of mind (ToM), or the ability to infer and predict the intentions, thoughts and beliefs of others, involves cognitive perspective taking (cognitive ToM/cToM) and understanding emotions (affective ToM/aToM). While behavioral evidence indicates that ToM is influenced by sex and age, no study has examined the influence of these variables on the neural correlates of cToM and aToM in late childhood/adolescence. Using fMRI with 35 typically-developing youths (aged 9–18 years, 12 males), we investigated the influence of sex and age on the neural correlates of cToM and aToM. We also examined how callous-unemotional traits, indexing a lack of empathy, were related to brain responses during aToM. Across both conditions, we found convergent activity in ToM network regions, such as superior temporal sulcus/temporoparietal junction (TPJ) and precuneus across males and females, but males recruited the left TPJ significantly more than females during cToM. During aToM, age was negatively correlated with brain responses in frontal, temporal and posterior midline regions, while callous-unemotional traits were positively correlated with right anterior insula responses. These results provide the first evidence in youth that sex influences the neural correlates of cToM, while age and callous-unemotional traits are specifically related to brain responses during aToM.

## Introduction

‘Theory of mind’ (ToM) is the ability to infer and predict others’ mental states, including intentions, thoughts and beliefs^1^. More recent conceptualizations of ToM are multidimensional^2^ and include understanding of others’ emotions as part of ToM^3,4^. A prominent model divides ToM into cognitive (*i.e*., inferring another’s beliefs/motivations) and affective (*i.e*., inferring what someone feels) components^5^. According to this model, affective ToM (aToM) requires the integration of cognitive ToM (cToM) and empathy, suggesting aToM might be a higher order and more complex component than cToM^5,6^.

**Figure 1:**
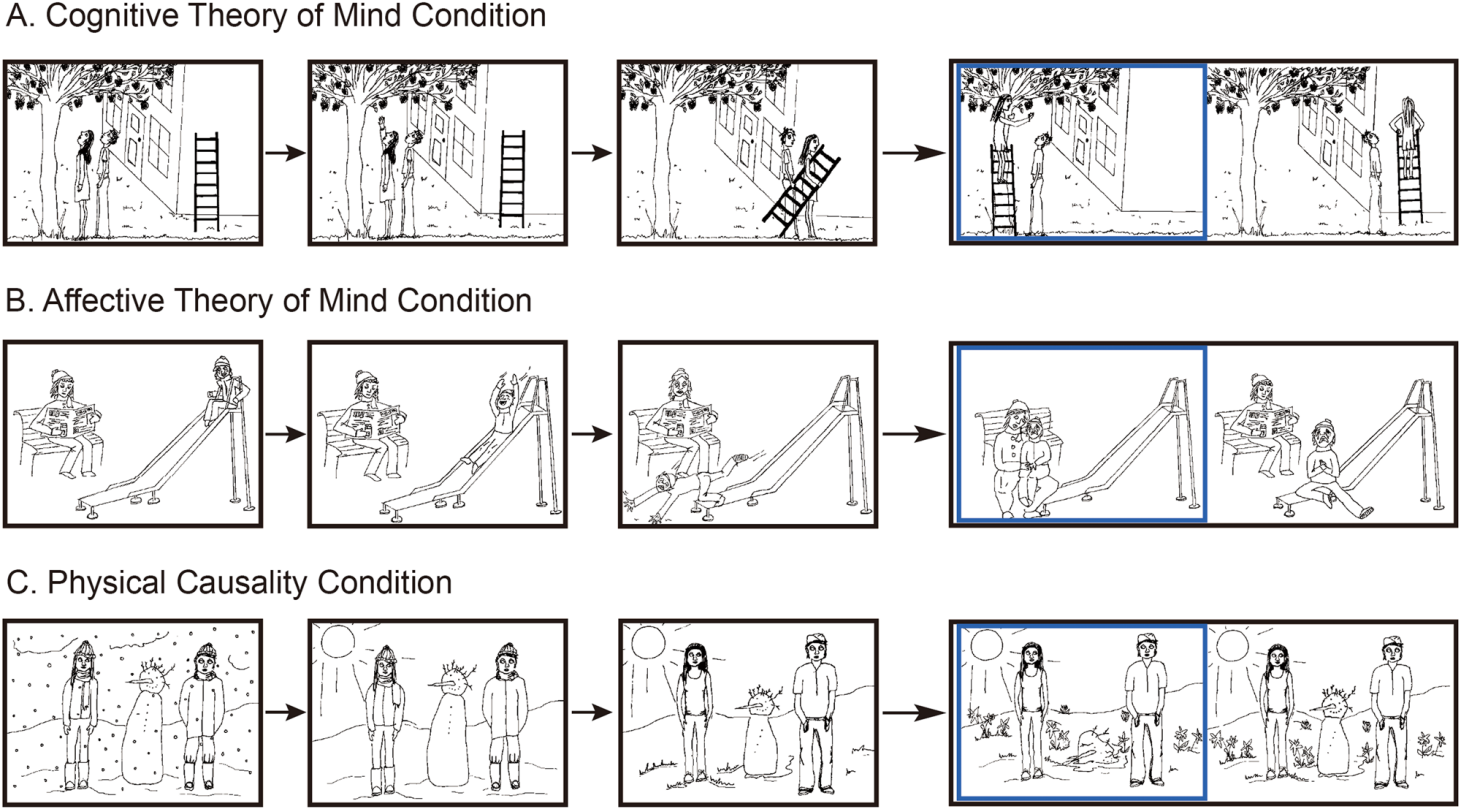
Examples of the cartoon vignette stimuli for (**A**) cognitive ToM, (**B**) affective ToM and (**C**) physical causality conditions. The frames of the story were sequentially displayed for 2 seconds each. The choice between two endings was displayed for 5 seconds. A blue frame highlighted the participant’s choice from the onset of the key press response until the end of the 5 seconds display. For illustrative purposes the correct answer is shown highlighted on the left of the display, although during the task the location of the correct answer was randomized.

Functional magnetic resonance imaging (fMRI) studies have delineated a ‘core brain network’ for ToM, including the medial prefrontal cortex, temporal-parietal junction (TPJ), posterior superior temporal sulcus (pSTS), posterior cingulate cortex (PCC) and precuneus^4,7,8^. To date, however, most studies have relied on adult samples despite evidence of significant development of the social brain between late childhood and late adolescence^9,10^. Using a cartoon-based vignette paradigm which includes separate conditions for cToM, aToM, and a physical causality (PC) control condition (see Fig. 1), Sebastian and colleagues examined the neural correlates of cToM and aTOM in 15 adolescent and 15 adult typically-developing males^6^. The results demonstrated that relative to the PC control condition, ToM conditions recruited the ‘core ToM network’; whereas the dorsolateral prefrontal cortex (dlPFC) was more involved during cToM, and ventromedial prefrontal cortex (vmPFC) and PCC activations were only observed during aToM, which is consistent with previous studies on adults^11–14^. Behaviorally, adolescent males, relative to adult males, made more errors on the aToM condition and reported lower levels of affective empathy trait. Finally, adolescents showed stronger activation in the vmPFC during aToM relative to adults^6^. Given these differences between adults and youths during ToM processing and the lack of research on youths, particularly in females, the first aim of the present study was to investigate the neural substrates underpinning cToM/aToM in a sample of typically-developing female youth.

Neuroimaging studies have also indicated possible sex differences in the neural correlates of ToM (see for review Christov-Moore *et al*.^15^). For example, using a statement re-appraisal task assumed to reflect cToM processing^16^, Veroude *et al*. reported that males activate bilateral inferior parietal cortex (e.g. TPJ), precuneus and PCC more than females^17^. Derntl *et al*. found that when inferring emotional response from a pictorial scenario, males activated TPJ more than females, while females activated inferior frontal gyrus (IFG), amygdala and the STS more than males^18^ (see also Schulte-Ruther *et al*.^19^). Taken together, these neuroimaging findings suggest that males activate TPJ more than females during cToM processing, while females recruit amygdala, IFG and STS more than males when inferring others’ emotional reactions. However, the current neuroimaging literature on sex differences in the correlates of ToM has several limitations. First, most previous studies examining the influence of sex have focused on cToM and aToM separately^15,20^. Second, participants in most of the existing neuroimaging studies were adults, meaning that far less is known about the influence of sex on the neural correlates of ToM in youths^21^. In this context, the second aim of the present study was to investigate the influence of sex on the neural correlates of cToM and aToM in youths.

Our third aim was to examine whether age as a continuous variable is associated with brain responses during cToM and aToM. Experimental work has shown that the development of cToM precedes that of aToM^22,23^. Consistent with these data, aToM performance has been found to be positively related to age and executive functions^24^ and there is evidence that adolescents recruit the vmPFC more than adults during aToM, suggesting a particularly protracted developmental trajectory of aToM^6^. There is thus substantial evidence that the social brain continues to develop between adolescence to adulthood^9,25^. However, to our knowledge, apart from one study^6^, no other fMRI study has investigated the relationship between age (as a continuous variable) and the neural correlates of cToM and aToM in the same study.

Finally, we examined if variation in callous-unemotional (CU) traits relates to neural responses during ToM. CU traits are a clinical construct reflecting a lack of empathy and guilt combined with a shallow affect and the callous use of others for one’s own gain^26^. Among antisocial youths, CU traits are negatively associated with performance and neural responses on affective, but not cognitive, ToM tasks^27–29^. Importantly, these traits are dimensionally distributed in the population and, as is the case in antisocial youths, are associated with behavioural and emotional problems and abnormal emotional responses in non-antisocial youths^26^. Given the lack of research on the neural correlates of CU traits in non-antisocial youths, we investigated whether neural responses during aToM were related to CU traits in typically-developing youths.

The current study aimed to replicate previous findings observed with males^6^ and tested whether these could be extended to female youth. The cartoon vignette paradigm combined with fMRI was used to investigate the neural correlates of cToM and aToM^6^. The impact of participants’ sex on the neural correlates of ToM was examined as well as associations with age and CU traits. We expected to replicate the patterns of neural activation observed during cToM and aToM reported by Sebastian and colleagues in female youth as well as in a combined-sex group^6^. In line with previous fMRI studies reporting sex differences during emotion processing^17–19^, we anticipated that males relative to females would show stronger involvement of the TPJ during cToM, while females would exhibit stronger involvement of the amygdala, STS and IFG specifically during aToM. Based on Sebastian and colleagues^6^, we hypothesized that age would be negatively correlated with vmPFC brain responses during aToM. Finally, given previous studies examining associations between CU traits in clinical samples and brain responses to empathy-eliciting stimuli and aToM^28,30,31^, and a recent review indicating that effects observed in community samples typically mirror those observed in clinical samples^32^, we hypothesized that, across females and males, CU traits would be negatively correlated with vmPFC, amygdala and anterior insula brain responses during aToM^28,33^.

## Results

### Questionnaire and behavioral results

As shown in Table 1, females scored higher on the affective empathy subscale of the GEM, but no sex differences were observed for cognitive and total empathy or CU traits. For the three conditions, mean reaction times (RTs) and percentage error rates were recorded for the two groups (Table 1). A 3 (condition: cToM, aToM, PC) by 2 (sex: female, male) mixed model ANOVA with mean RT as the dependent variable showed marginal main effects of condition [F(2,66) = 3.13, *P* = 0.050, η^2^ = 0.58], indicating that RTs tended to be shorter in the PC than the cToM condition (*P* = 0.07, Bonferroni corrected). No significant main effect of sex (*P* = 0.60, η^2^ = 0.08) or interactions (*P* = 0.43, η^2^ = 0.19) were found. A condition-by-sex mixed model ANOVA for percentage error rate revealed no significant main effect of condition (*P* = 0.17, η^2^ = 0.32) or sex (*P* = 0.98, η^2^ = 0.05). There was, however, a marginal interaction (*P* = 0.053, η^2^ = 0.53), showing numerically that females made more errors in the aToM, while males made more errors in the cToM and PC tasks, but post-hoc analyses (Bonferroni corrected) showed no significant sex differences across aToM (*P* = 0.16), cToM (*P* = 0.12) and PC (*P* = 0.51) conditions.

**Table 1:** Means and standard deviations for the demographic, questionnaire, and behavioral data for the cartoon Theory of Mind (ToM) task, presented by Condition and Group.

	Female	Male	*t*	*P*	Cohen’s *d*
Age in years (s.d.)	14.96(2.74)	15.00(2.04)	−0.05	0.96	0.02
IQ (s.d.)	100.13(8.45)	98.75(6.94)	0.49	0.63	0.18
***ICU scores (s.d.)***					
Total scores	14.52(8.89)	17.50(7.78)	−0.98	0.33	0.36
Callous	3.65(3.80)	4.00(2.17)	−0.29	0.77	0.11
Uncaring	7.09(5.09)	9.00(4.24)	−1.11	0.27	0.41
Unemotional	3.78(2.41)	4.50(2.97)	−0.77	0.45	0.27
***GEM scores (s.d.)***					
Total scores	17.12(24.39)	30.67(17.79)	−1.70	0.10	0.63
Cognitive	3.87(10.88)	10.00(9.77)	−1.64	0.11	0.59
Affective	6.39(9.55)	−3.42(15.14)	2.35	**0.03**	0.78
***Mean RT (s.d.)***					
Affective ToM	2124(477)	2277(444)	−0.92	0.36	0.33
Cognitive ToM	2202(478)	2286(390)	−0.52	0.61	0.19
Physical Causality	2101(470)	2099(367)	0.02	0.99	<0.01
***Percent errors (s.d.)***					
Affective ToM	13.91(14.38)	7.50(7.54)	1.44	0.16	0.56
Cognitive ToM	5.22(9.47)	10.00(6.03)	−1.58	0.12	0.60
Physical Causality	5.65(6.62)	7.50(9.65)	−0.67	0.51	0.22

s.d. = standard deviation, IQ = intelligence quotient, ICU = Inventory of Callous-Unemotional Traits, GEM = Griffith Empathy Measure, RT = reaction time.

### fMRI results

#### Main effects of condition

**Figure 2:**
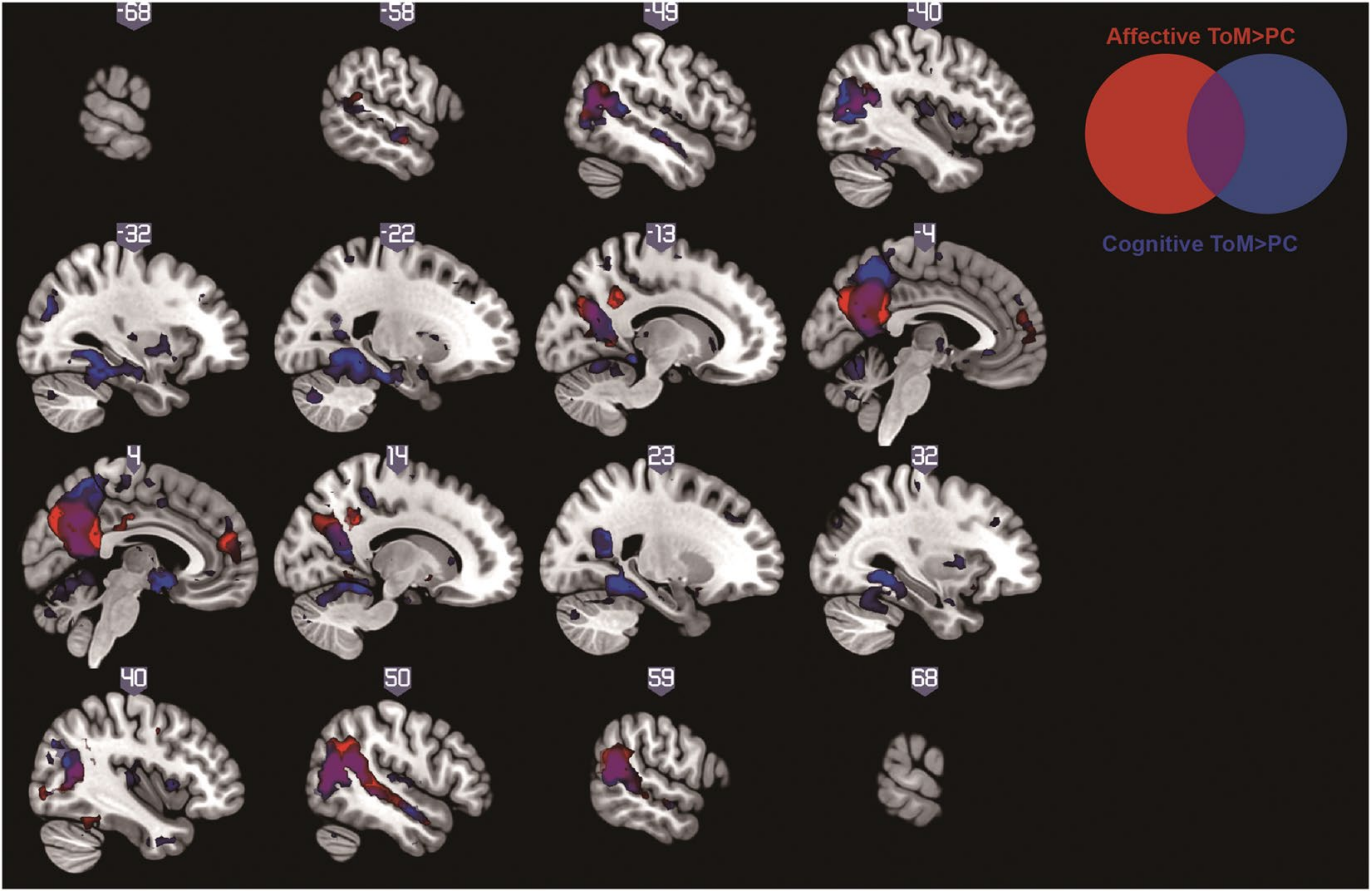
Brain regions that were significantly more active for affective ToM (in red) or cognitive ToM (in blue) than the physical causality condition in the female group. The sagittal view is shown at a threshold of *P* < 0.001, uncorrected for display purposes. For both contrasts, significant clusters were seen in the posterior temporal sulcus/temporoparietal junction, temporal poles and precuneus.

Table 2 shows regions that reached a cluster-level significance of *P* < 0.05, familywise error correction (FWE), for the four contrasts of interest, in female youth. The results are largely consistent with the findings of Sebastian *et al*.^6,28^. For the aToM > PC contrast, we found significant activation in bilateral STS/TPJ and precuneus/PCC (Fig. 2). For the cToM > PC contrast, significant activation was found in the bilateral precuneus/PCC, STS/TPJ and parahippocampal gyrus. There was a significant difference between aToM and cToM (affective > cognitive) in bilateral PCC activation. For the contrast cToM > aToM, significant activation was observed in bilateral middle occipital gyrus, extending into culmen (see Supplementary Tables S1, S2 and Fig. S1 for the results for the combined-sex group and in males alone).

**Table 2:** Regions showing a main effect for each contrast at *P* < 0.05 with cluster-level FWE correction in the female group.

Brain region	BA	L/R	Peak voxel			*k*	*Z*	*P*-value
			x	y	z			
***Affective ToM*** > ***PC***								
STS/TPJ ext. middle temporal cortex	22	R	57	−51	15	401	5.53	<0.001
	39	R	57	−57	24		5.22	
	39	R	51	−63	12		5.18	
Precuneus/PCC	30	R	6	−48	18	572	5.33	<0.001
	7	L	−3	−60	33		5.26	
	31	L	−6	−54	27		5.24	
STS/TPJ ext. middle temporal cortex	19	L	−42	−78	9	91	4.21	0.004
	39	L	−45	−63	18		3.89	
	13	L	−42	−48	15		3.82	
***Cognitive ToM > PC***								
STS/TPJ ext. middle temporal cortex	40	R	54	−48	18	274	4.72	<0.001
	39	R	45	−63	18		4.62	
	22	R	60	−54	12		4.56	
Precuneus/PCC	31	L	−15	−60	18	107	4.59	0.003
	30	L	−18	−54	9		3.54	
	29	L	−9	−51	6		3.51	
Parahippocampal gyrus ext. culmen	37	L	−30	−57	−6	75	4.42	0.013
	19	L	−30	−48	−6		4.16	
	18	L	−27	−45	−15		3.87	
PCC	23	R	9	−54	15	110	4.33	0.002
	30	R	27	−57	12		3.90	
STS/TPJ ext. middle temporal cortex	37	L	−39	−63	9	205	4.33	<0.001
	39	L	−45	−72	24		4.20	
	19	L	−42	−72	6		4.05	
Parahippocampal gyrus ext. fusiform gyrus	37	R	36	−42	−9	65	4.32	0.023
	37	R	24	−48	−9		3.96	
Precuneus	7	—	0	−48	54	115	3.60	0.002
	7	—	0	−60	36		3.41	
	7	—	0	−60	51		3.35	
***Affective ToM > Cognitive ToM***								
PCC	31	R	6	−54	27	58	4.23	0.019
	30	R	6	−48	21		4.21	
	31	L	−6	−54	24		3.46	
***Cognitive ToM > Affective ToM***								
Middle occipital gyrus ext. culmen	—	L	−24	−45	−21	1020	5.70	<0.001
	—	R	27	−45	−12		5.34	
	19	L	−30	−81	18		5.12	

BA = Brodmann area, ToM = Theory of mind, PC = Physical causality, PCC = posterior cingulate cortex, STS = superior temporal sulcus, TPJ = temporal parietal junction, L/R = left/right, peak voxel = MNI *xyz* co-ordinates, *k* = cluster size. Where more than one BA is shown, the peak voxel falls in the first BA, but the cluster extends to include the others listed (as indicated by ‘ext.’).

#### Sex differences

**Figure 3:**
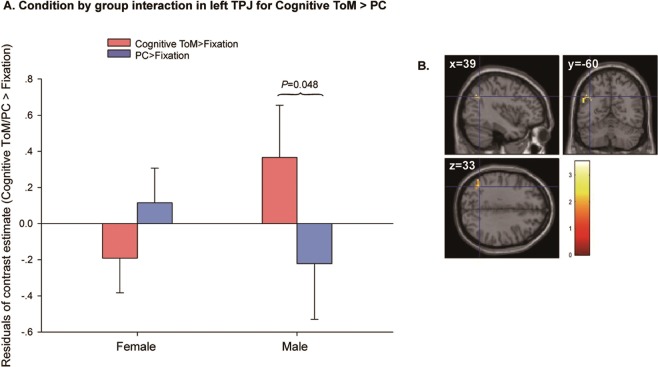
Sex difference in brain responses in the left temporoparietal junction for the contrast cognitive ToM > physical causality (PC): (**A**) Residual graph showing the nature of the interaction in the peak voxel [39, −60, 33] after regressing out age and IQ. Males showed a greater response in this region during cognitive ToM than during PC, while no differences between those conditions were observed in the females; (**B**) the cluster in left temporoparietal junction showing the main effect of group in cognitive ToM > PC contrast. The colored bar represents *t*-statistics. The statistical parametric map is displayed at a threshold of *P* < 0.001 uncorrected for display purposes.

For the cToM > PC contrast, a sex-by-condition interaction was observed in left TPJ (Fig. 3; peak voxel for this interaction = [−39, −60, 33], k = 38, z = 3.19; *P* = 0.033, FWE- small volume correction [SVC]). Post-hoc paired *t*-tests revealed that the interaction was driven by males showing greater TPJ brain responses during the cToM than the PC condition [*t*(11) = 2.23, *P* = 0.048], whereas no difference between conditions was observed in females [*t*(22) = −1.60, *P* = 0.12]. For the other contrasts of interest, no additional interactions with sex were observed at a whole-brain level and within the regions-of-interest (ROIs).

#### Correlations with age and CU traits across females and males

Age: For the aToM > PC contrast, there was a significant negative relationship between age and responses in right precuneus, extending into PCC (Table 3). For the aToM > cToM contrast, significant negative correlations between age and bilateral dlPFC, right PCC, middle frontal cortex and right TPJ responses were observed. No other correlations with age were observed for the other contrasts.

**Table 3:** Regions showing a correlation between the age and brain responses for the contrasts of interest across all participants at *P* < 0.05 with cluster-level FWE correction.

Brain Region	BA	L/R	Peak voxel			*k*	*Z*	*P*-value
			x	y	z			
**Affective ToM > PC**								
Precuneus/PCC	29, 31	R	9	−45	15	54	4.61	0.042
**Affective ToM > Cognitive ToM**								
dlPFC	8	R	24	27	36	131	4.67	0.001
	8	L	−24	15	48	52	4.47	0.043
PCC	31	R	6	−54	27	370	4.34	<0.001
Middle frontal cortex ext. precentral gyrus	6	R	39	3	48	66	4.33	0.018
TPJ ext. supra marginal gyrus	40	R	54	−48	33	297	4.29	<0.001

BA = Brodmann area, ToM = Theory of mind, PC = physical causality, PCC = posterior cingulate cortex, TPJ = temporal parietal junction, dlPFC = dorsal lateral prefrontal cortex, L/R = left/right, peak voxel = MNI *xyz* co-ordinates, *k* = cluster size.

**Figure 4:**
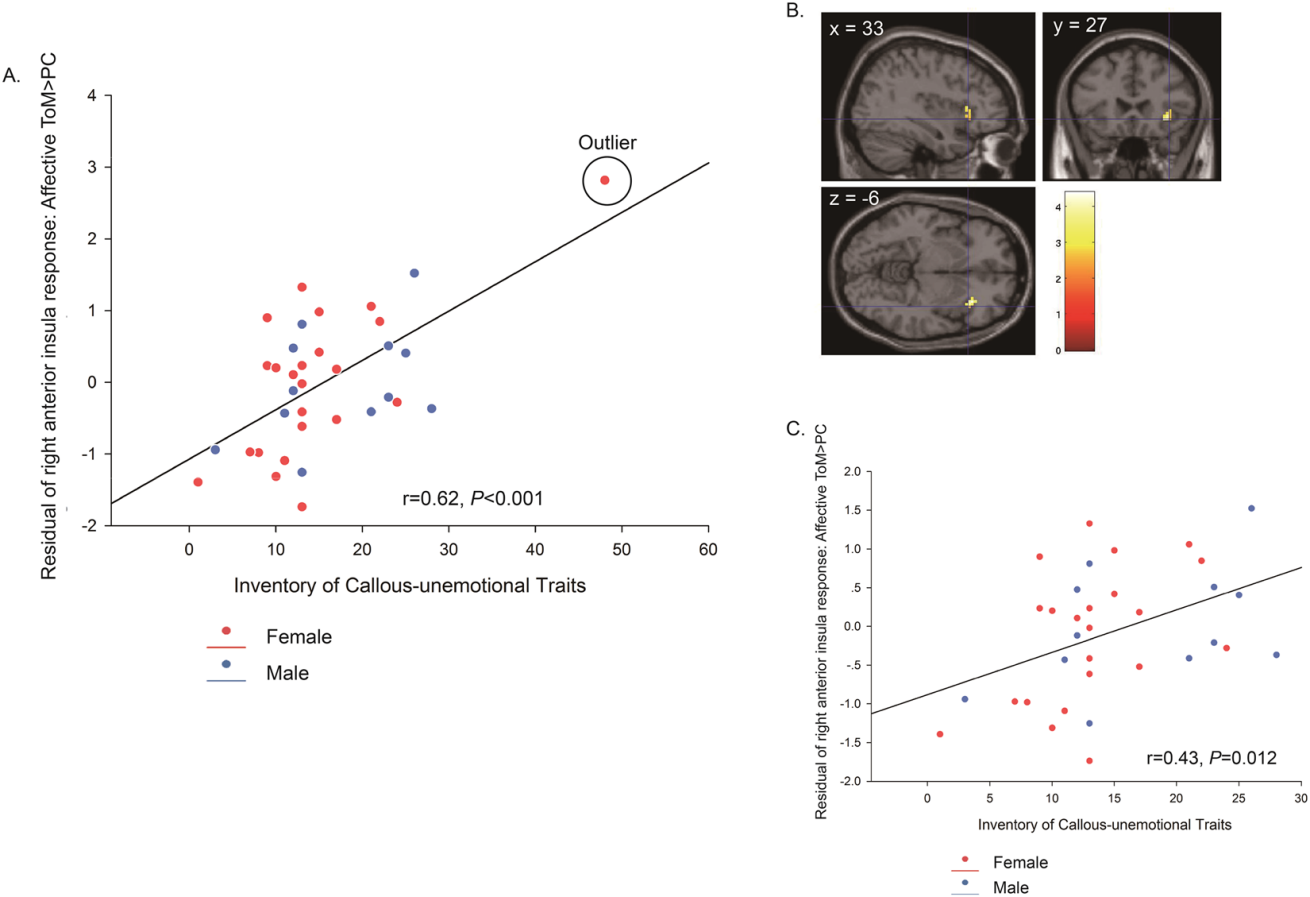
Correlation between brain responses in the right anterior insula for affective ToM > physical causality (PC) and CU traits (r = 0.64, *P* < 0.001): (**A**) Residual plot showing correlation between CU traits and right anterior insula responses in the peak voxel [33, 27, −6] after regressing out age, sex and IQ; (**B**) the cluster in right anterior insula showing the significant association with CU traits in affective ToM > PC contrast; (**C**) the residual plot without the outlier in female group. The colored bar represents *t*-statistics. The statistical parametric map is displayed at a threshold of *P* < 0.001 for display purposes.

CU traits: For the aToM > PC contrast, there was a positive correlation between CU traits scores and right anterior insula responses (Fig. 4; peak voxel = [33, 27, −6], k = 4, z = 3.82; *P* = 0.010, FWE-SVC). After removing the outlier in the female group, the correlation remained significant in both SPM and SPSS analyses (Fig. 4C). No other regions showed an association with CU traits.

## Discussion

The present study investigated the neural correlates of cognitive and affective ToM in typically-developing youths and examined the influence of sex, age and CU traits. First, in line with our hypothesis, and consistent with Sebastian *et al*.^6^, in female children and adolescents we identified an overlapping ‘core brain network’ for both cToM and aToM conditions, which included bilateral STS/TPJ, PCC and precuneus^4,7,8^. Partially supporting our hypothesis and replicating Sebastian and colleagues’ findings, dlPFC responses for the cToM contrast (relative to aToM) were observed across sexes. However, no significant vmPFC activation was observed in females or across sexes for the aToM contrast (relative to PC). Second, partially supporting our predictions, males showed increased brain responses in the left TPJ for the cToM contrast (relative to PC), but no sex differences were observed in the amygdala, the anterior insula and the IFG for aToM. Third, partially supporting our hypothesis, age was negatively correlated with brain responses mostly in frontal (but not vmPFC as hypothesized), temporal and posterior midline regions only when contrasting aToM with cToM conditions. Fourth, against our predictions, CU traits were positively correlated with right anterior insula responses during aToM only, but no association with amygdala responses was identified.

Overall, the pattern of brain responses to cToM and aToM conditions relative to PC was similar across conditions, with activation in the bilateral pSTS/TPJ and precuneus (extending into PCC). These areas have been suggested to make up the classical ‘core brain network’ for ToM^34–36^. Our results in females only and across sexes are broadly consistent with those of Sebastian *et al*.’s study and support their conclusion that cToM and aToM may recruit overlapping brain regions^6^. Consistent with our hypothesis, and replicating Sebastian *et al*.’s results^6^, cToM relative to PC activated bilateral dlPFC across sexes [dlPFC activation was seen in females, but at a lower threshold (peak voxel = [27, 6, 57], k = 85, z = 4.19, P = 0.16)], which has also been identified to be related to cToM in adults^12,13^. However, against our prediction, and in contrast to Sebastian *et al*.^6^, at our chosen statistical threshold we did not detect activation in the vmPFC for aToM relative to PC in females alone or across sexes. This might be due to a number of reasons, such as a less homogenous sample (*i.e*., mixed sex) spanning a wider age range. Notwithstanding, a cluster in the vmPFC was identified when using the coordinates from Sebastian and colleagues (peak voxel = [6, 51, 18], k = 27, z = 4.06; FWE-SVC), thus corroborating the involvement of this region in aToM. Overall, the present study replicates and extends Sebastian *et al*.’s findings by identifying overlapping (as well as distinct) neural substrates underpinning cToM/aToM in a female-only sample and a mixed-sex sample spanning the period from late childhood to late adolescence.

Although males and females appeared to share a common ‘core brain network’ supporting ToM, one sex difference in brain responses emerged for cToM whereby males showed greater activity in the left TPJ than females. Evidence from previous studies has suggested an important role for the TPJ in tracking what others’ think and for responding to mental state information from toddlerhood through to adulthood^34,37,38^. For example, a recent near-infrared spectroscopy study reported that other than prefrontal regions, infants aged around 7-months recruited TPJ more when others’ belief regarding the location of the object was false compared to when the belief was true^38^, thus highlighting the fundamental role of the TPJ in ToM as early as infancy. Further evidence comes from lesion data showing that the left TPJ is a necessary brain area for reasoning about others’ beliefs^39^. Our results are also consistent with a previous study reporting that adult males exhibited increased bilateral TPJ activation compared to females when making appraisals of self and other, as well as reflected self-appraisal^17^. Given that we found no sex differences in accuracy and reaction time for cToM, we speculate that the observed sex difference in the neural correlates of cToM (greater TPJ recruitment in males) might reflect a ‘compensatory’ effect. This interpretation is consistent with previous behavioral studies indicating poorer cToM performance in males^40^.

Consistent with the developmental differences (adolescents > adults) reported in the vmPFC by Sebastian *et al*. for aToM^6^, age exhibited negative correlations with brain responses, mostly in frontal (although not vmPFC), temporal and posterior midline regions. This was, however, only true for the aToM > cToM contrast. This result is in line with behavioral and fMRI evidence that development of aToM is more protracted than that of cToM^6,22,23^. Given that aToM is thought to require the integration of cToM and empathy^5,41^, the negative correlations we observed between age and brain responses to the contrast aToM > cToM are consistent with several previous fMRI studies (but see Greimel *et al*.^42^), which have identified reduced responses with increasing age across the lifespan in tasks indexing empathy for negative and positive emotions^43–46^. The exact mechanism underlying this association still remains poorly understood, but could reflect different processes that are not mutually exclusive. These include pruning^10^ (but see Chen *et al*.^44^ and Riva *et al*.^46^ who found no association with grey matter changes), different cognitive strategies, increased automaticity of processes with age^6^, or increased ‘neural efficiency’ consequent to increased practice^47^. Interestingly, age was negatively associated with activation of the bilateral dlPFC during aToM, an area shown to be involved in cToM in adults^12–14^. Taken together, these findings suggest that aToM recruits bilateral dlPFC less with increasing age, while cToM consistently relies on dlPFC throughout the lifespan.

Interestingly, our prediction in relation to CU traits and brain responses to aToM was not supported; CU traits correlated with right anterior insula responses during aToM relative to PC, but the correlation was positive, not negative as hypothesized. Against our prediction, no association with amygdala responses was observed. The anterior insula is a core brain region for processing aversive information^48^, empathy for pain^21,49^, and processing affective and physiological states^48,50^. The right anterior insula, in particular, has been specifically implicated in the affective-perceptual form of empathy^21^, which could account for the observed association between brain responses in this region during aToM and CU traits indexing poor empathy. Indeed, because the aToM condition requires the integration of both cToM and empathy, and the fact that we did not observe any association between CU traits and responses during the cToM condition, we speculate that the observed correlation in right anterior insula is driven by the empathy component of the aToM condition. As such, our results are consistent with those of two previous studies focusing on antisocial youths, which have reported a correlation between CU traits and brain responses in the anterior insula using empathy-eliciting stimuli^30,31^. However, in contrast to these studies, we observed a positive association between CU traits and insula responses. Based on our interpretation of the negative association between age and brain responses to the same condition, the positive correlation with CU traits could reflect ‘increased effort’ to perform the task. Interestingly, a recent study from our group on typically-developing youths has reported a positive association between CU traits and grey matter volume in the anterior insula in males, but not females^51^. Finally, in contrast to Sebastian *et al*. where CU traits were negatively correlated with amygdala responses to aToM relative to cToM^28^, we found no such correlation.

Our study had some limitations that should be noted. Notwithstanding the power analyses, our sample size was relatively small in particular when considering the number of males for our examination of sex effects, so our results in relation to sex differences should be considered preliminary and interpreted with care until replicated in larger samples. Despite this, we were able to replicate the main effects of condition reported by Sebastian *et al*.^6^, thus providing additional support for, and extending our understanding of, the neural correlates of aToM and cToM in late childhood and adolescence. In addition, the complexity of the processes examined in the different conditions and of the task and its contrasts means that it is unclear which exact subcomponent(s) of aToM/cToM and empathy might be driving the effects that we and others have reported^6,28,29^. Methodologically, several strategies were adopted to reduce nuisance artefacts, including visual inspection of the data, realignment, co-registration using an anatomical scan, removing participants with excessive head motion (one participant removed), adding six estimated realignment parameters as nuisance regressors as well as adding extra regressors to account for a small number of corrupted images resulting from excessive motion. However, we acknowledge that more advanced methods exist to deal with motion-based noise^52^ and the task-related fMRI data might also be partly influenced by a few non-neural sources of variability due to the intrinsic features of blood oxygen-level-dependent fMRI^52^.

In conclusion, we were able to replicate the classical neural substrates underlying cToM and aToM and extend previous findings to a female-only sample and a mixed-sex sample of typically-developing youth spanning late childhood to late adolescence. To our knowledge, this is the first fMRI study to investigate the influence of sex on the neural correlates of cToM and aToM in youth. Our results suggest that male youth recruit the left TPJ more during cToM than do female youth. Finally, the association between age and brain responses during aToM suggests increased neural efficiency with advancing age, whereas the association between CU traits and brain responses during aToM might reflect increased effort in those with higher levels of CU traits.

## Methods

### Participants

Twenty-three typically-developing girls and thirteen typically-developing males (age range = 9–18 years) were recruited. However, due to excessive head motion (over 10% scans displaying >3 mm displacement), one male was subsequently excluded, leaving 12 males in the final sample. Our sample size is consistent with previous fMRI studies on ToM in young adults as reported in two recent meta-analyses^8,53^. A power analysis for replicating the main effects of condition in female group used the weakest *Z*-stats of the peak from reported results by Sebastian *et al*. (*Z* = 3.91, observed for the comparison between of cToM > aToM in the left dlPFC, Table 2)^6^. The *Z*-value was converted to *t*-value using the reported sample size [*t*(29) = 4.54]. We then computed the Cohen’s *d*_*z*_ = *t*/(n^0.5^)^54^, which resulted in a minimum expected Cohen’s *d*_*z*_ = 0.83. In G*power, using paired sample *t*-test with an alpha of 0.05, to account for the multiple ROIs, a sample of 18 will be associated with 95% power. This means that the sample of 23 female provides a well-powered study to be able to replicate the previously reported results. A power analysis for exploring sex differences used the *Z* value of the group comparison from reported results by Sebastian *et al*. (*Z* = 3.54, observed for the comparison between adolescents and adults in the left vmPFC)^6^. The *Z* value was converted to *t* value using the reported sample size [*t*(29) = 4.00]. We then computed the Cohen’s *d* = *t* × (1/n_1_ + 1/n_2_)^0.5^, which resulted in a Cohen’s *d* = 1.46^54^. In G*power, using two groups *t*-test with an alpha of 0.05, to account for the multiple ROIs, a sample of 11 participants in each group will be associated with 95% power. This means that the sample of 23 females and 12 males provides a well-powered study to be able to investigate group differences. Finally, our total sample size of 35 participants is also comparable to previous fMRI studies that have examined brain response-personality correlations^55,56^ and consistent with previous simulation work indicating that an average sample size of 18.25 is needed for this type of analyses^57^.

The sample was recruited from mainstream primary and secondary schools, youth groups and community centers in Birmingham (UK), as part of the FemNAT-CD project^58^. Only a small number of participants (mostly typically-developing youths) completed the cartoon fMRI task, which was only used at Birmingham and the last (bonus) task in our MRI protocol, hence the small number of participants compared to the wider FemNAT-CD study. According to the FemNAT-CD recruitment protocol, all participants and their parents/caregivers completed the Schedule for Affective Disorders and Schizophrenia for School-Age Children-Present and Lifetime version interview (K-SADS-PL)^59^. Based on this interview, we ascertained that participants were typically-developing. The exclusion criteria included an estimated IQ below 70; inability to speak or understand English; any monogenetic disorder; any genetic syndrome; any chronic or acute neurological disorder; autism spectrum disorder, schizophrenia, bipolar disorder or any current mental health disorder or learning disorders besides dyslexia. Participants with past mental health disorders, excluding disruptive behaviour disorders and psychosis, were included if they were in remission (no symptoms for 12 months; no participants included in this report were in remission). Ethical approval was granted by the National Health Service (NHS) Research Ethics Committee (13/WM/0483). All individuals under the age of 16 included in the study were required to provide consent and parental consent for participation. As per UK ethical guidelines, adolescents aged 16 and above could consent for themselves without the need for parental consent. However, where parents/legal guardians of those youths were available, we also obtained consent from them. Finally, parents always provided informed consent for their own participation.

CU traits were assessed with the 24-item parent-report version of the Inventory of Callous-Unemotional Traits (ICU)^60^, which contains three subscales: callousness (e.g., ‘I do not care who I hurt to get what I want’), uncaring (e.g., ‘I always try my best’, reverse-scored), and unemotional (e.g., ‘I do not show my emotions to others’). Participants’ cognitive and affective empathy abilities were assessed using the 23-item parent version of the Griffith Empathy Measure (GEM)^61^; items scored from −4 (strongly disagree) to 4 (strongly agree). As shown in Table 1, there was no sex difference in age [*t*(33) = −0.05, *P* = 0.96] nor in full-scale IQ [*t*(33) = 0.49, *P* = 0.63] as measured by the two-subtest version of the Wechsler Abbreviated Scale of Intelligence^62^.

### Experimental task

We employed a well-validated block-design fMRI task previously used in typically-developing adolescents and adults and in youths with conduct problems and autism spectrum disorders (Fig. 1)^6,28,29^. The task included 30 static cartoon vignette stimuli: 10 each for the cToM, aToM, and PC conditions. Four sequential frames were involved in each cartoon. The first screen (1 second) displayed an instruction ‘What happens next?’. This was followed by three sequentially presented story frames (2 seconds each), depicting two people in everyday scenarios. The final screen (5 seconds) showed a choice of two possible endings for the cartoon. During this time participants were asked to decide the appropriate ending and make their choice using an MR-compatible button-box. There was an inter-stimulus interval of 1 second between trials. Each trial lasted 13 seconds in total.

Thirty cartoons were presented in sets of six, with a 13-second fixation period between sets. Each cartoon was presented once only. The six cartoons in each set included two yoked cartoons from each condition. The order of the cToM, aToM and PC cartoon pairs in each set was randomized for each participant. cToM trials required understanding behaviour based on intentions to select the correct ending (e.g., using a ladder to help reach apples on a tree). aToM cartoons required understanding behaviour based on empathy and emotion (e.g., comforting an injured child). PC cartoons involved an understanding of physical cause and effect reasoning (e.g., sunshine melting a snowman).

### fMRI data acquisition

A 3 T Philips Achieva MRI scanner at the Birmingham University Imaging Centre was used to acquire a T2*-weighted echo planar imaging (EPI) volumes using a 32-channel head coil. EPI data were acquired in a single run of 8 minutes, with 184 task volumes and 5 dummy volumes. Acquisition parameters were: 41 slices; TE = 30 ms; TR = 2500 ms; matrix size = 64 × 64; voxel size = 3 × 3 × 3 mm^3^; flip angle = 83°; field of view = 192 mm; slice thickness = 2 mm. In addition, a high resolution, sagittal, 3D T1-weighted scan with an in-plane resolution of 1 × 1 × 1 mm^3^ and lasting 5.5 minutes, was acquired for normalization of the EPI data. Acquisition parameters were: 192 slices; TE = 3.7 ms; TI = 900 ms; TR = 8 ms; flip angle = 9° and matrix size = 256 × 256.

### fMRI data pre-processing and analysis

Imaging data were pre-processed using SPM12 (www.fil.ion.ucl.ac.uk/spm) in Matlab R2017a. The first five volumes were removed to allow for T1 equilibrium effects, leaving 184 volumes per participant. The EPI data were first realigned and co-registered to the high-resolution T1-weighted scan. Next, the Template-O-Matic toolbox was used to create standardized a priori tissue probability maps (TPMs) based on the age and the sex of the 35 participants^63^. The high-resolution T1-weighted scans were segmented with reference to the TPMs into grey matter and white matter images, based on a multi-channel approach implemented with the Computational Anatomy Toolbox 12 (CAT12)^64^. The segmented grey and white matter images were then used to generate a template using the Diffeomorphic Anatomical Registration Through Exponentiated Lie Algebra toolbox (DARTEL)^65^. This template was used to normalize the grey and white matter segmented images by iteratively warping the images into a common space using non-linear registration. Finally, the DARTEL template and EPI images were normalized to Montreal Neurological Institute (MNI) standard space^66^ with a voxel size of 3 × 3 × 3 mm^3^. Data were smoothed using a Gaussian kernel of full width at half maximum resolution of 6 × 6 × 6 mm^3^ to account for residual inter-subject differences and to comply with the continuity assumption of random field theory^67^.

The data were analyzed using a participant-specific general linear model (GLM) with a blocked-design analysis procedure to compare the neural activity associated with cToM, aToM and PC. The time series of 184 volumes was deconstructed into seven blocks: presentation of each of the three cartoon conditions (11 seconds duration), the periods of fixation (13 seconds) and instructions for each condition (‘What happens next?’; 1 second). The regressors were modelled as boxcar functions and convolved with a canonical hemodynamic response function. The six realignment parameters were modelled as effects of no interest in order to account for any variance due to head movement. For seven participants extra regressors were added at the first-level to model a small number of corrupted images resulting from excessive motion. Consistent with Sebastian *et al*. (2012), these images (ranging from 0.5–2.7% of total acquired volumes across the seven participants; M = 1.8%) were removed and the adjacent images interpolated to avoid distortion of the between-subjects mask. A high-pass filter (cutoff = 128 sec) and AR(1) correction for serial autocorrelation were applied during the least mean square estimation of this GLM.

At the first-level, four contrasts of interest were created for each participant: 1) cToM > PC, 2) aToM > PC, 3) cToM > aToM and 4) aToM > cToM. Contrast images were then carried forward to second-level analyses. Except when examining age effects, age and IQ were included as covariates of no interest in all second-level analyses. To test whether the results replicate previously reported results for males and can be extended to females, we first analyzed each sex group separately. To assess sex differences, we used sex (female, male) as the between-subjects factor. The effects of age (controlling for sex and IQ) and CU traits (controlling for age, sex and IQ) were assessed using continuous variables that were added to second level models (*i.e*., 2 multiple regressions). For all analyses, the grey matter TPM was used as an explicit grey matter mask, thresholded at 0.3 to create a binary mask.

At a whole-brain level, results are reported at *P* < 0.05, FWE-cluster level corrected unless specified, in line with Sebastian *et al*.^6^. For region-of-interest analyses, consistent with Sebastian *et al*.^6^, reported results are those that survived SVC at *P* < 0.05, FWE corrected with a 10 mm sphere centered on peak co-ordinates taken from the main effects analysis (*i.e*., one-sample *t*-tests) across all participants (an orthogonal contrast). Second, for the analyses involving sex, age and CU traits, we also report effects within the amygdala, the vmPFC, the anterior insula and the IFG to be consistent with fMRI studies on empathy examining the influence of those variables within the above regions of interest^18,21,28,30,31,33^. Based on Sebastian *et al*.’s work using this task^6,28^, the amygdala mask was defined bilaterally using 10 mm spheres centered on the peak coordinates (left = [−24, −5, −13]; right = [24, −5, −13]), the vmPFC mask was taken from peak coordinates (left = [−8, 54, 0]; right = [10, 50, 22]) while the anterior insula mask consisted of bilateral spheres centered on the peak coordinates (left = [−36, 26, 1]; right = [36, 26, 1]). The IFG mask was defined by peak coordinates (left = [−54, 30, 2]; right = [54, 30, 2]) from Schulte-Rüther *et al*. reporting sex differences in evaluating others’ emotion^19^. Where significant group effects, associations with CU traits or age were observed, contrast estimates from the peak voxel (owing to smoothing, contrast estimates in the peak voxel are a weighted average of the surrounding voxels) were extracted to assess direction of effects, plot the results and run correlational analyses in SPSS 19.0 (SPSS, Chicago, IL).

## Supplementary information

Supplementary material

## Data Availability

The datasets generated and/or analyzed during the current study are available from the corresponding author on reasonable request.
