# Supplementary material for: Neural correlates of theory of mind in typically-developing youth: Influence of sex, age and callous-unemotional traits
Source: Sci Rep. 2019 Nov 7;9:16216. doi: 10.1038/s41598-019-52261-y (PMC6838181; doi:10.1038/s41598-019-52261-y)
Supplement: Supplementary file 1 — Supplementary material [file 41598_2019_52261_MOESM1_ESM.doc]

**Neural correlates of theory of mind in typically-developing youth: Influence of sex, age and callous-unemotional traits**

Yidian Gao^1,2+^, Jack C. Rogers^2,3+^, Ruth Pauli^2,3^, Roberta Clanton^2,3^, Rosalind Baker^2,3^, Philippa Birch^2,3^, Lisandra Ferreira^2,3^, Abigail Brown^2,3^, Christine M. Freitag^4^, Graeme Fairchild^5^, Pia Rotshtein^2,3^, and Stephane A. De Brito^2,3^*

^1^ Medical Psychological Institute, Second Xiangya Hospital, Central South University, Changsha, Hunan, China

^2^ Centre for Human Brain Health, School of Psychology, University of Birmingham, Birmingham, UK

^3^ Institute for Mental Health, School of Psychology, University of Birmingham, Birmingham, UK

^4^ Department of Child and Adolescent Psychiatry, Psychosomatics and Psychotherapy, University Hospital Frankfurt, Goethe University, Frankfurt am Main, Germany

^5^ Department of Psychology, University of Bath, Bath, UK

+ These authors contributed equally to this work

*Corresponding author:

Stephane De Brito, Centre for Human Brain Health, School of Psychology, Robert Aitken Building, University of Birmingham, B15 2TT, UK. Email: s.a.debrito@bham.ac.uk

**Supplementary Tables**

Table S1. Regions showing a main effect for each contrast at *P*<0.05 with cluster-level FWE correction in the mixed-gender group

|  |  |  | Peak voxel | | |  |  |  |
| --- | --- | --- | --- | --- | --- | --- | --- | --- |
| Brain Region | BA | L/R | x | y | z | *k* | *Z* | *P*-value |
| ***Affective ToM > PC*** |  |  |  |  |  |  |  |  |
| Precuneus/PCC | 30 | R | 6 | -51 | 18 | 937 | 6.81 | <.001 |
|  | 31 | L | -9 | -54 | 30 |  |  |  |
|  | 31 | R | 3 | -53 | 33 |  |  |  |
| STS/TPJ ext. middle temporal cortex | 40 | R | 54 | -48 | 18 | 602 | 6.42 | <.001 |
|  | 19 | R | 54 | -63 | 12 |  |  |  |
|  | 39 | R | 45 | -54 | 12 |  |  |  |
| STS/TPJ ext. middle temporal cortex | 39 | L | -45 | -54 | 12 | 225 | 4.89 | 0.013 |
|  | 39 | L | -51 | -63 | 21 |  |  |  |
| ***Cognitive ToM > PC*** |  |  |  |  |  |  |  |  |
| Precuneus/PCC | 31 | L | -18 | -60 | 18 | 982 | 5.89 | <.001 |
|  | 30 | R | 21 | -57 | 18 |  |  |  |
|  | 7 | R | 6 | -51 | 36 |  |  |  |
| STS/TPJ ext. middle temporal cortex | 22 | R | 60 | -57 | 12 | 481 | 5.81 | <.001 |
|  | 39 | R | 42 | -60 | 21 |  |  |  |
| STS/TPJ | 39 | L | -45 | -72 | 24 | 366 | 4.87 | <.001 |
|  | 22 | L | -42 | -54 | 18 |  |  |  |
| Parahippocampal gyrus ext. fusiform gyrus | 37 | R | 36 | -42 | -9 | 131 | 4.75 | 0.001 |
| Parahippocampal gyrus ext. fusiform gyrus | 37 | L | -30 | -42 | -15 | 142 | 4.66 | 0.001 |
| ***Affective ToM > Cognitive ToM*** |  |  |  |  |  |  |  |  |
| PCC | 31 | R | 6 | -54 | 27 | 92 | 4.52 | 0.004 |
|  | 31 | L | -6 | -54 | 21 |  |  |  |
| ***Cognitive ToM > Affective ToM*** |  |  |  |  |  |  |  |  |
| Parahippocampal gyrus ext. lingual gyrus | 37 | L | -27 | -45 | -18 | 700 | 6.15 | <.001 |
|  | 18 | L | -6 | -84 | -9 |  |  |  |
| Parahippocampal gyrus | 35 | R | 30 | -30 | -21 | 209 | 5.62 | <.001 |
| Middle occipital gyrus ext. cuneus | 19 | L | -30 | -78 | 21 | 628 | 5.57 | <.001 |
|  | 19 | L | -30 | -78 | 30 |  |  |  |
| dlPFC | 8 | L | -24 | 6 | 54 | 78 | 5.16 | 0.009 |
| Superior occipital gyrus | 19 | R | 33 | -81 | 27 | 81 | 4.35 | 0.008 |
| dlPFC | 6 | R | 27 | 6 | 54 | 68 | 4.26 | 0.016 |

BA=Brodmann area, ToM=Theory of mind, PC=Physical causality, PCC=posterior cingulate cortex, STS=superior temporal sulcus, TPJ=temporal parietal junction, dlPFC=dorsal lateral prefrontal cortex, L/R=left/right, peak voxel=MNI *xyz* co-ordinates, *k*=cluster size. Where more than one BA is shown, the peak voxel falls in the first BA, but the cluster extends to include the others listed (as indicated by ‘ext.’).

Table S2. Regions showing a main effect for each contrast at *P*<0.05 with cluster-level FWE correction and at *P*<.001 (uncorrected, k≥5) in the male group

|  |  |  | Peak voxel | | |  |  | *P*-FWE corrected | *P* uncorrected |
| --- | --- | --- | --- | --- | --- | --- | --- | --- | --- |
| Brain Region | BA | L/R | x | y | z | *k* | z |
| ***Affective ToM > PC*** |  |  |  |  |  |  |  |  |  |
| Middle temporal cortex | 21 | R | 54 | 9 | -21 | 57 | 4.94 | 0.005 | < 0.001 |
|  | 21 | R | 48 | 3 | -24 |  | 3.98 |  |  |
|  | 21 | R | 60 | 0 | -15 |  | 3.66 |  |  |
| Posterior cingulate cortex ext. precuneus | 30 | L | 0 | -51 | 21 | 209 | 4.73 | < 0.001 | < 0.001 |
|  | 29 | L | -3 | -57 | 12 |  | 4.51 |  |  |
|  | 31 | L | 0 | -63 | 24 |  | 3.96 |  |  |
| STS/TPJ ext. middle temporal cortex | 22 | R | 63 | -48 | 15 | 72 | 4.13 | 0.001 | < 0.001 |
|  | 13 | R | 48 | -45 | 15 |  | 3.91 |  |  |
|  | 22 | R | 60 | -36 | 6 |  | 3.36 |  |  |
| TPJ | 39 | L | -54 | -57 | 12 | 25 | 3.87 | 0.12 | < 0.001 |
|  | 39 | L | -51 | -63 | 21 |  | 3.36 |  |  |
| ***Cognitive ToM > PC*** |  |  |  |  |  |  |  |  |  |
| Precuneus/PCC | 31 | L | -18 | -66 | 21 | 29 | 3.85 | 0.15 | < 0.001 |
| PCC | 31 | R | 21 | -60 | 18 | 20 | 3.85 | 0.32 | < 0.001 |
| STS/TPJ | 22 | R | 63 | -48 | 15 | 31 | 3.72 | 0.13 | < 0.001 |
|  | 22 | R | 60 | -57 | 15 |  | 3.72 |  | < 0.001 |
|  | 13 | R | 54 | -39 | 21 |  | 3.37 |  | < 0.001 |
| Precuneus/PCC | 31 | R | 6 | -51 | 33 | 32 | 3.61 | 0.12 | < 0.001 |
|  | 31 | R | 9 | -45 | 39 |  | 3.40 |  | < 0.001 |
|  | 31 | L | -6 | -48 | 36 |  | 3.22 |  | < 0.001 |
| Precuneus | 7 | L | -9 | -54 | 48 | 14 | 3.55 | 0.54 | < 0.001 |
| TPJ | 39 | L | -48 | -69 | 27 | 6 | 3.48 | 0.90 | < 0.001 |
|  |  | L | -39 | -66 | 30 |  | 3.32 |  | < 0.001 |
| ***Cognitive ToM > Affective ToM*** |  |  |  |  |  |  |  |  |  |
| Culmen | - | L | -33 | -33 | -24 | 6 | 4.18 | 0.89 | < 0.001 |
| Precuneus ext. superior occipital gyrus | 19 | L | -27 | -78 | 36 | 21 | 3.99 | 0.26 | < 0.001 |
|  | 19 | L | -36 | -75 | 24 |  | 3.50 |  | < 0.001 |
| Insula | 13 | R | 30 | -30 | 21 | 5 | 3.68 | 0.93 | < 0.001 |
| Fusiform Gyrus | 37 | L | -33 | -48 | -9 | 19 | 3.67 | 0.31 | < 0.001 |
|  | 37 | L | -33 | -36 | -12 |  | 3.46 |  | < 0.001 |
| Precuneus | 7 | L | -9 | -72 | 51 | 7 | 3.39 | 0.85 | < 0.001 |
|  |  | L | -24 | -69 | 48 |  | 3.17 |  | < 0.001 |

BA=Brodmann area, ToM=Theory of mind, PC=Physical causality, PCC=posterior cingulate cortex, STS=superior temporal sulcus, TPJ=temporal parietal junction, L/R=left/right, peak voxel=MNI *xyz* co-ordinates, *k*=cluster size. Where more than one BA is shown, the peak voxel falls in the first BA, but the cluster extends to include the others listed (as indicated by ‘ext.’).

**Supplementary Figure**


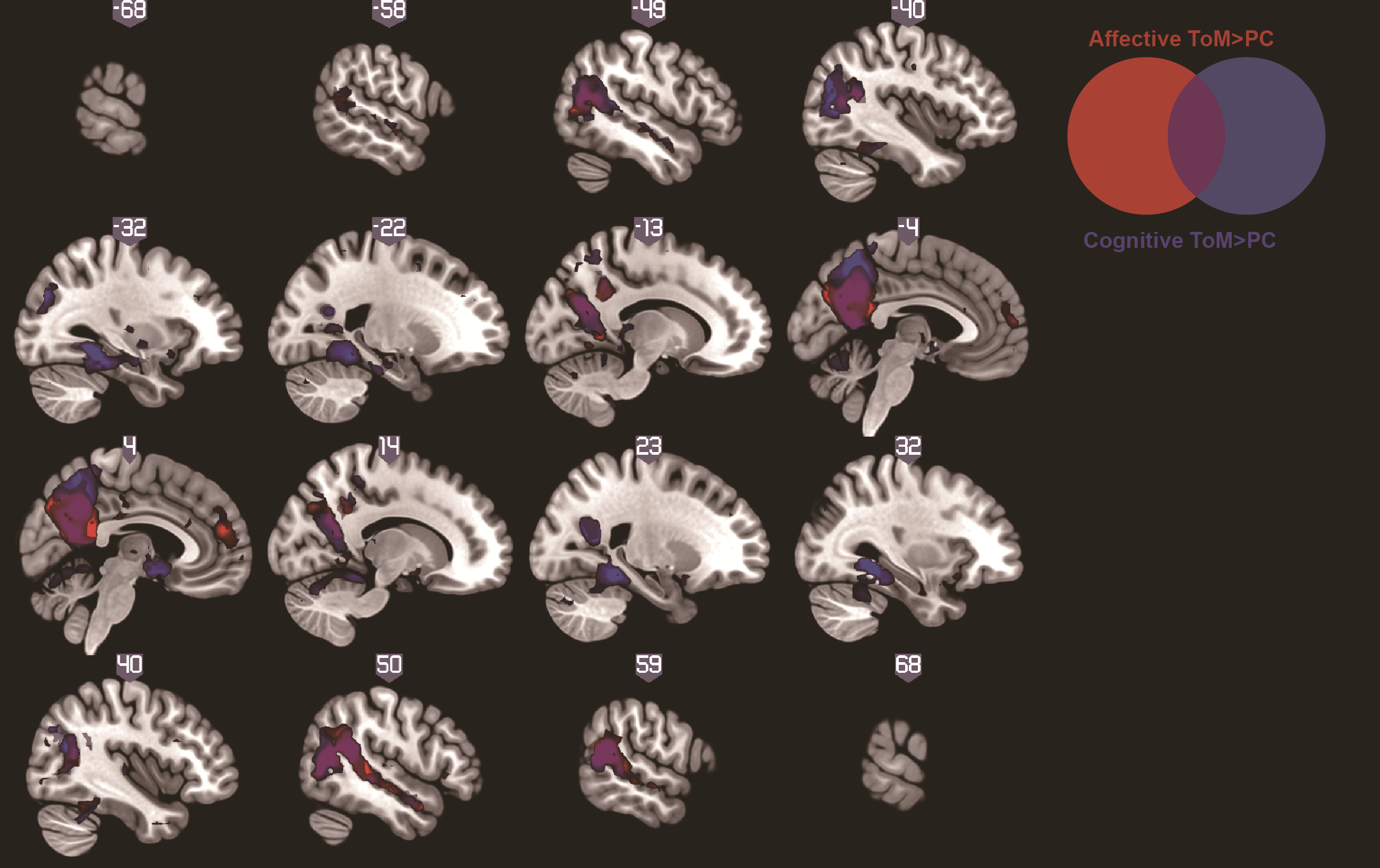


Figure S1. Brain regions that were significantly more active for affective ToM (in red) or cognitive ToM (in blue) than the physical causality condition in the mixed-gender group. Sagittal view is shown at a threshold of *P*<.001, uncorrected for display purposes. For both contrasts, significant clusters were seen in the posterior temporal sulcus/temporoparietal junction, temporal poles and precuneus.
